# Diagnostic Utility of IGF2BP1 and Its Targets as Potential Biomarkers in ETV6-RUNX1 Positive B-Cell Acute Lymphoblastic Leukemia

**DOI:** 10.3389/fonc.2021.588101

**Published:** 2021-02-23

**Authors:** Gunjan Sharma, Elza Boby, Thakur Nidhi, Ayushi Jain, Jay Singh, Archna Singh, Parthaprasad Chattopadhyay, Sameer Bakhshi, Anita Chopra, Jayanth Kumar Palanichamy

**Affiliations:** ^1^ Department of Biochemistry, All India Institute of Medical Sciences, New Delhi, India; ^2^ Department of Laboratory Oncology, Dr. B.R. Ambedkar Institute Rotary Cancer Hospital, All India Institute of Medical Sciences, New Delhi, India; ^3^ Department of Medical Oncology, Dr. B.R. Ambedkar Institute Rotary Cancer Hospital, All India Institute of Medical Sciences, New Delhi, India

**Keywords:** *IGF2BP1*, *EGFL7*, *ETV6-RUNX1* translocation, B-ALL, receiver operating characteristic curve (ROC)

## Abstract

Around 85% of childhood Acute Lymphoblastic Leukemia (ALL) are of B-cell origin and characterized by the presence of different translocations including *BCR-ABL1*, *ETV6-RUNX1*, *E2A-PBX1*, and *MLL* fusion proteins. The current clinical investigations used to identify *ETV6-RUNX1* translocation include FISH and fusion transcript specific PCR. In the current study we assessed the utility of *IGF2BP1*, an oncofetal RNA binding protein, that is over expressed specifically in *ETV6-RUNX1* translocation positive B-ALL to be used as a diagnostic marker in the clinic. Further, public transcriptomic and Crosslinked Immunoprecipitation (CLIP) datasets were analyzed to identify the putative targets of IGF2BP1. We also studied the utility of using the mRNA expression of two such targets, *MYC* and *EGFL7* as potential diagnostic markers separately or in conjunction with *IGF2BP1*. We observed that the expression of *IGF2BP1* alone measured by RT-qPCR is highly sensitive and specific to be used as a potential biomarker for the presence of *ETV6-RUNX1* translocation in future.

## Introduction

B-cell acute lymphoblastic leukemia (B-ALL) is the commonest childhood cancer in the pediatric population which originates from the propagation of cytogenetically altered and molecularly abnormal B-lymphocyte progenitor cells (1). The defective progenitor cells, leukemic blasts, exhibit uncontrolled proliferation, get accumulated in the bone marrow and further infiltrate into various extramedullary sites, including spleen, liver, lymph node, thymus, and gonads (2). About 50% of B-ALL tumors exhibit genetic rearrangements among which translocations are most common (3). A set of sentinel genetic lesions, mostly *BCR/ABL1*, *ETV6/RUNX1*, *E2A/PBX1*, and *MLL* rearrangements, have been well recognized in B-ALL. From Western literature, we see that *ETV6/RUNX1* is the commonest translocation seen in B-ALL which decreases with increasing age. The frequency of *BCR/ABL1* is very low in childhood ALL (4). However, the translocation profile seen in the Asian population is quite different. *ETV6-RUNX1* is present at a lower frequency while *BCR-ABL1* shows a higher prevalence (5–9).

*ETV6-RUNX1* fusion is formed by the fusion of the first five exons of *ETV6* proximally to almost the entire *RUNX1* gene depending upon the breakpoint region (10). Both ETV6 and RUNX1 are transcription factors and play an important role in haematopoiesis (11) with ETV6 known to be a strong repressor (12). The chimeric *ETV6/RUNX1* transcription factor retains the essential pointed N terminal protein-protein interaction domain of ETV6 and the DNA-binding and transcriptional regulatory sequences of RUNX1 (13). *ETV6-RUNX1* translocation has been seen in 1%–5% of normal new-borns (14–16) which is around hundred times the number who develop overt clinical leukemia (10, 16). This demonstrates that *ETV6-RUNX1* translocation alone is insufficient for leukemogenesis and another genetic event (e.g., deletion of the other ETV6 allele) is required (3).

*In situ* hybridization is an important technique in the field of cytogenetics to study the presence of different chromosomal abnormalities (17). With the improvement in this technique, the identification of different translocations is now done by Fluorescence-in Situ Hybridisation (FISH) (metaphase or interphase), using fluorescent probes (18–20) which are expensive, time consuming and labor-intensive (21). Despite having several advantages, the major limiting factor from the diagnostic point of view is the availability of patient sample. Further, the process may fail occasionally due to unsuccessful culture. Alternatively, RT-PCR for fusion transcripts is also used but has variable results in comparison to FISH (22) along with a high rate of false-positive results (15) as well as false-negative results due to RNA instability (14). The success rate of these techniques depends upon the stability of the fusion transcripts, hybridization of the probe (Fluorescent probe or primer) to the DNA which further depends on the copy number of the fusion transcripts in question.

To overcome these limitations, we tried to use RT-qPCR of specific genes as an alternative to diagnose the presence of the *ETV6-RUNX1* translocation. Overexpression of the RNA binding protein (RBP), Insulin like Growth Factor 2 mRNA Binding Protein 1 (IGF2BP1) has been reported previously in *ETV6-RUNX1* positive B-ALL patients (23). IGF2BP1 is an oncofetal protein overexpressed in epithelial cancers including colorectal and breast cancers and is known to bind oncogenic mRNAs and increase their stability (24). In this study, we have demonstrated the utility of IGF2BP1 expression in B-ALL patient bone marrow samples to diagnose the presence of the *ETV6-RUNX1* translocation. Since IGF2BP1 is an RNA binding protein (RBP), we have also used data mining of multiple high throughput public datasets to identify its binding targets and have tested the utility of the expression of two of these targets, *EGFL7* and *MYC*, to diagnose the translocation. We conclude that all of these have the potential to be good candidates for identifying the presence of the translocation with *IGF2BP1* being the best among them.

## Material and Methods

### Patient Samples

Inclusion/Exclusion criteria: Newly diagnosed B-ALL patients (diagnosis established by morphology, immunophenotyping, and molecular genetics), age from 0 to 18 years, and written informed consent of parents obtained prior patient enrolment. Patients treated elsewhere initially or ALL developing secondary to another malignancy were excluded from the study.

From 261 enrolled naïve B-ALL patients, we were able to collect sufficient patient bone marrow (BM) samples from 114 patients at the time of routine diagnosis from March 2016 to August 2019 at BR Ambedkar Institute Rotary Cancer Hospital at AIIMS, New Delhi after obtaining ethical clearance from the Institutional Ethics Committee and informed consent from a guardian, and assent from children above 7 years of age. To increase the power of the study, we have also included 29 [1 altered cytogenetics, 13 other known translocations (11 *BCR-ABL1*, 1 *E2A-PBX1*, 1 *MLL*), 15 *ETV6-RUNX1* positive] archival samples (bone marrow samples collected before March 2016) for this study. Due to the limitation with the sample volume collected, we were not able to study all the genes in all the patient samples.

### Cell Culture

Reh (*ATCC* CRL-8286) (*ETV6-RUNX1* translocation positive B lymphoblastic cell line) cells were cultured in RPMI1640 (Himedia AL199S) with 10% Fetal Bovine Serum (Gibco) and 1% antibiotics (Pen-Strep, Gibco) as recommended by ATCC at 37°C and 5% CO_2_.

### Patient Sample Preparation

The bone marrow sample collected was subjected to RBC Lysis Buffer for 30 min at 4°C. The samples were centrifuged at 1,500 g for 10 min at 4°C and washed with 1X Phosphate Buffer Saline (Gibco) followed by centrifugation. The pellet obtained was resuspended in 1 ml of TRIzol (Thermo Fisher) followed by RNA isolation.

### RNA Isolation

For every, 1 ml TRIzol sample, 200 µl chloroform was added and mixed vigorously for 10 sec followed by 15 min incubation at room temperature. After incubation, samples were centrifuged at 12,000g for 15 min at 4°C to allow layers to separate. After centrifugation, the samples were taken out carefully and the upper aqueous layer was collected without disturbing the interface in a fresh tube. To the separated sample, 0.5 ml of isopropanol (Thermo) was added and incubated for 10 min at room temperature. After incubation, the samples were centrifuged at 12,000g for 10 min at 4°C to precipitate the RNA. The supernatant was discarded and the precipitated RNA was washed with 70% ethanol (Thermo) at 7,500g for 10 min. The supernatant was discarded and the pellet was air dried to remove the residual ethanol, dissolved in nuclease free water (Qiagen) and stored at −80°C till further use.

### Real Time PCR

cDNA was prepared from the isolated RNA using RevertAid Reverse Transcriptase (Thermo Fisher) 10 U/µl, Random Decamers 5 µM/reaction (Sigma) and Ribolock (Thermo Fisher) 1 U/µl with 500–1,000 ng of RNA. Real time PCR (qPCR) was done (35 cycles, 95°C for 15 s, 60°C for 15 s, 72°C for 30 s) with Syto9 (Invitrogen) using specific primers for *IGF2BP1*, *MYC*, *ETV6-RUNX1*, *and EGFL7* (**Table 1**). The qPCR reactions were done in technical triplicates for all genes. The expression was normalized using housekeeping genes *RNA Polymerase II* and *PPIA.* ΔΔCt method was used to compare the gene expression (25).

**Table 1:** Primer sequences used for real time PCR.

Gene	Forward primer (5’➔3’)	Reverse primer (5’➔3’)
*IGF2BP1*	TTACTGGGGCTGCTCCCTAT	TTCGGGTGGTGCAATCTTGA
*MYC*	TACAACACCCGAGCAAGGAC	AGCTAACGTTGAGGGGCATC
*ETV6-RUNX1*	TGCACCCTCTGATCCTGAAC	AACGCCTCGCTCATCTTGC
*RNA Polymerase II*	CATCAAGAGAGTCCAGTTCGG	CCCTCAGTCGTCTCTGGGTA
*PPIA*	CACCGTGTTCTTCGACATTG	TTCTGCTGTCTTTGGGACCT

### Statistics

All experiments were done in triplicates. Relative expression was compared between different groups using Mann Whitney (two groups)/Kruskal-Wallis (more than two groups) statistical tests using GraphPad Prism software version 5. ROC curve was made using SPSS software version 20. A p-value of <0.05 was considered to be significant.

## Results

### Descriptive Statistics

**Figure 1:**
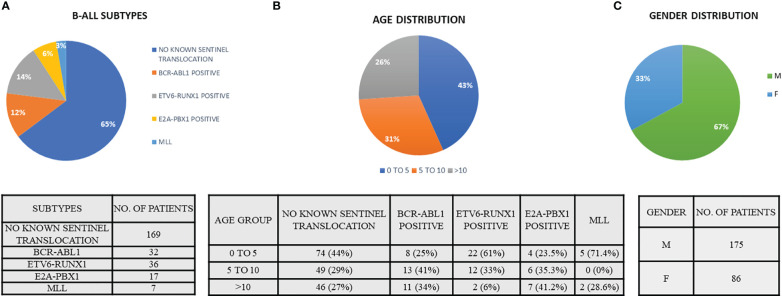
Demographic data indicating the **(A)** B-ALL subtypes, **(B)** age distribution of B-ALL patients, and **(C)** gender distribution. (Patient data reported from March 2016 to August 2019).

A total of 261 pediatric B-ALL patients were registered in the BRAIRCH OPD, AIIMS from March 2016 to August 2019. Out of these patients, 64.7% cases belonged to the “No known sentinel translocation” group, followed by *ETV6-RUNX1* (13.8%), *BCR-ABL1* (12.3%), *E2A-PBX1* (6.5%), and *MLL* gene translocation (2.7%). A lower incidence of *ETV6-RUNX1* (13%) incidence was noted as compared to the Western population (25%) in our patient cohort which is in concordance with the literature published for the Asian population (6–8). Similarly, a higher incidence of pediatric *BCR-ABL1* (11.5%) translocation was also observed as reported by other studies (7–9, 26, 27) (**Figure 1A**, **Table 2**). A male preponderance (M: F = 2:1) was observed in the pediatric population with a median age of 4 years (**Figures 1B, C** and **Table 2**).

**Table 2:** Demographic data of B-ALL patients recruited from March 2016–August 2019.

TRANSLOCATION	AGE			Total
	0–5	5–10	>10	
No known Sentinel Translocations	74	49	46	169
BCR-ABL1	8	13	11	32
ETV6-RUNX1	22	12	2	36
E2A-PBX1	4	6	7	17
MLL	5	0	2	7
Total	113	80	68	261
**Gender**	**Frequency**	**Percent**
Female	86	33.0
Male	175	67.0
Total	261	100.0

### RT-qPCR Analysis for *IGF2BP1* Expression

**Figure 2:**
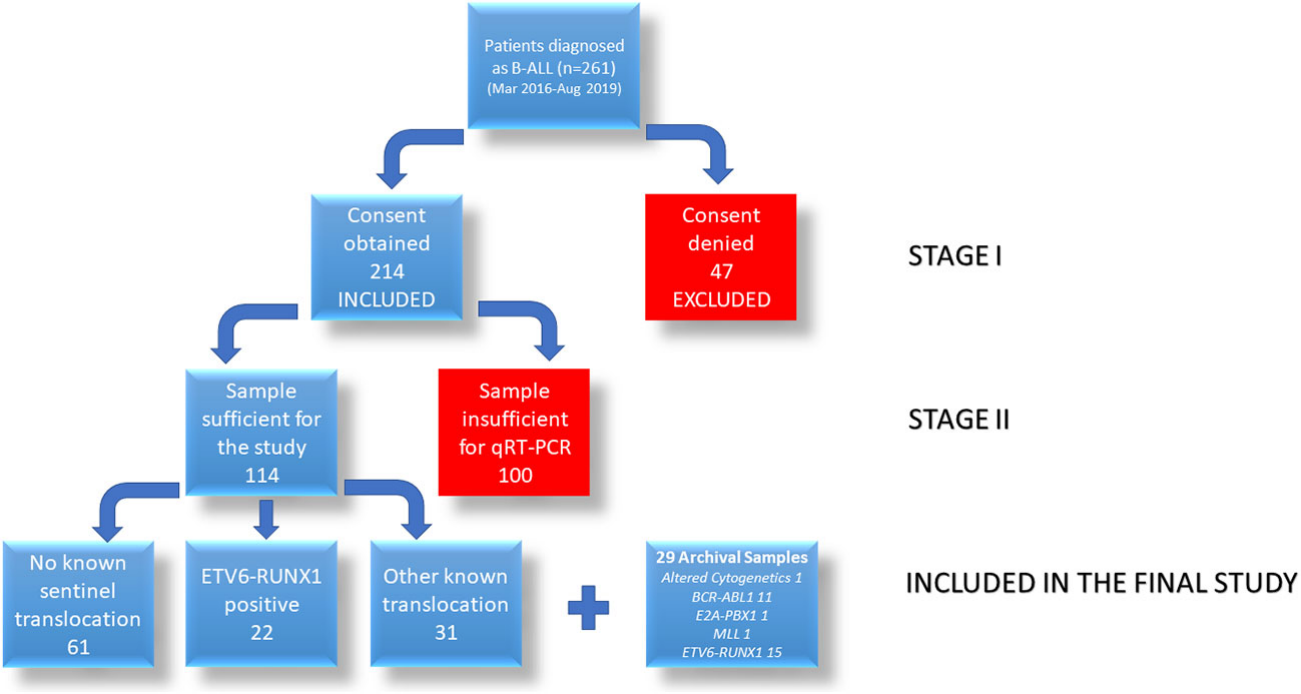
Flowchart showing the recruitment of patients for the study.

From the 261 patients, sufficient bone marrow sample for further analysis could be obtained for 114 patients (**Figure 2**). These patients were diagnosed on the basis of morphology, cytochemistry and immunophenotyping. *IGF2BP1* mRNA expression was studied by real time PCR using cDNA prepared from BM mononuclear cells using TRIzol. We also included 29 archival patient samples to increase the power of the study. The final analysis was done on 143 patient samples including 37 *ETV6-RUNX1* positive samples, 44 samples with other translocations (*E2A-PBX1* (n = 15), *BCR-ABL1* (n = 24), *MLL* (n = 5) fusion proteins), and 62 samples with no known translocations. The last group also included 13 patients with altered cytogenetics [Hyper diploidy (n = 8), hypodiploidy (n = 1), chromosomal deletions (n = 1) (chr 6q21)]. The relative expression of *IGF2BP1* was normalized using *RNA Pol II* and *PPIA*. Similar results were obtained using either of the housekeeping genes.

**Figure 3:**
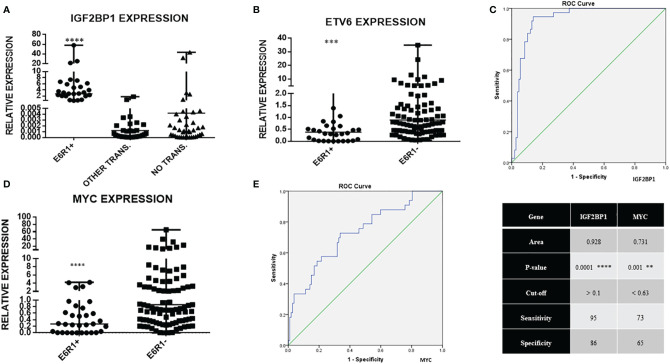
Real time expression data showing **(A)** IGF2BP1 overexpression in ETV6-RUNX1 translocation positive patients (N = 37), patients with other translocations (N = 44, BCR-ABL1, E2A-PBX1, MLL), and patients with no known translocation (N = 62, includes patients with altered cytogenetics, N = 13); **(B)** ETV6 expression in ETV6-RUNX1 translocation positive (N = 32) and translocation negative (N = 101) patients; **(C)** ROC curve for IGF2BP1 comparing it to FISH/RT-PCR for ETV6-RUNX1; **(D)** MYC expression in ETV6-RUNX1 translocation positive (N = 34) and ETV6-RUNX1 translocation negative (N = 106) patients, and **(E)** ROC curve for MYC expression. ****p < 0.0001; ***p < 0.001, **p < 0.01. ETV6-RUNX1 positive is denoted as E6R1+ and ETV6-RUNX1 negative as E6R1-.

*IGF2BP1* mRNA expression was significantly higher in *ETV6-RUNX1* positive B-ALL (n = 37), (median value = 2.74) compared to the negative samples (n = 106), (median value = 0.0022) (p < 0.0001, > 1,000 fold overexpression) (**Figure 3A**). This group also showed a significant downregulation in wild type *ETV6* expression levels, which is concordant with the loss of the other wild type *ETV6* allele reported in this subtype of B-ALL (28) (**Figure 3B**). The expression of *IGF2BP1* appeared to be specific to the *ETV6-RUNX1* translocation positive sub-group. Interestingly, some patients (12/62) in the “No known sentinel translocations” group had a high expression of *IGF2BP1*. In addition, 4/12 of them had altered cytogenetics. One of them had hyperploidy (53–54 chromosomes) including chromosome 17 which harbors the *IGF2BP1* gene. One patient showed extra signals for 21q (*RUNX1* locus, iamp21) on FISH and one patient’s sample was positive for extra signals for both 12p as well as 21q. The other 8 were cytogenetically normal.

A new subtype of B-ALL known as “*ETV6-RUNX1*-like leukemia” has been identified recently based on a gene expression profile similar to that of *ETV6-RUNX1* translocation positive leukemia in the absence of the translocation. This subtype also reported a functional loss of ETV6 in the form of mutation or deletions (29, 30). A lower relative expression of *ETV6* was noticeable in the patients with high *IGF2BP1* expression in the “No known sentinel translocation” group. It is interesting to speculate that these few patients might belong to the newly identified *ETV6-RUNX1* like leukemia subgroup. Loss or perturbation of the ETV6 or its regulatory loci might be triggering an *ETV6-RUNX1*-like leukemia in these patients.

### Testing the Efficacy of *IGF2BP1* as a Diagnostic Marker

**Figure 4:**
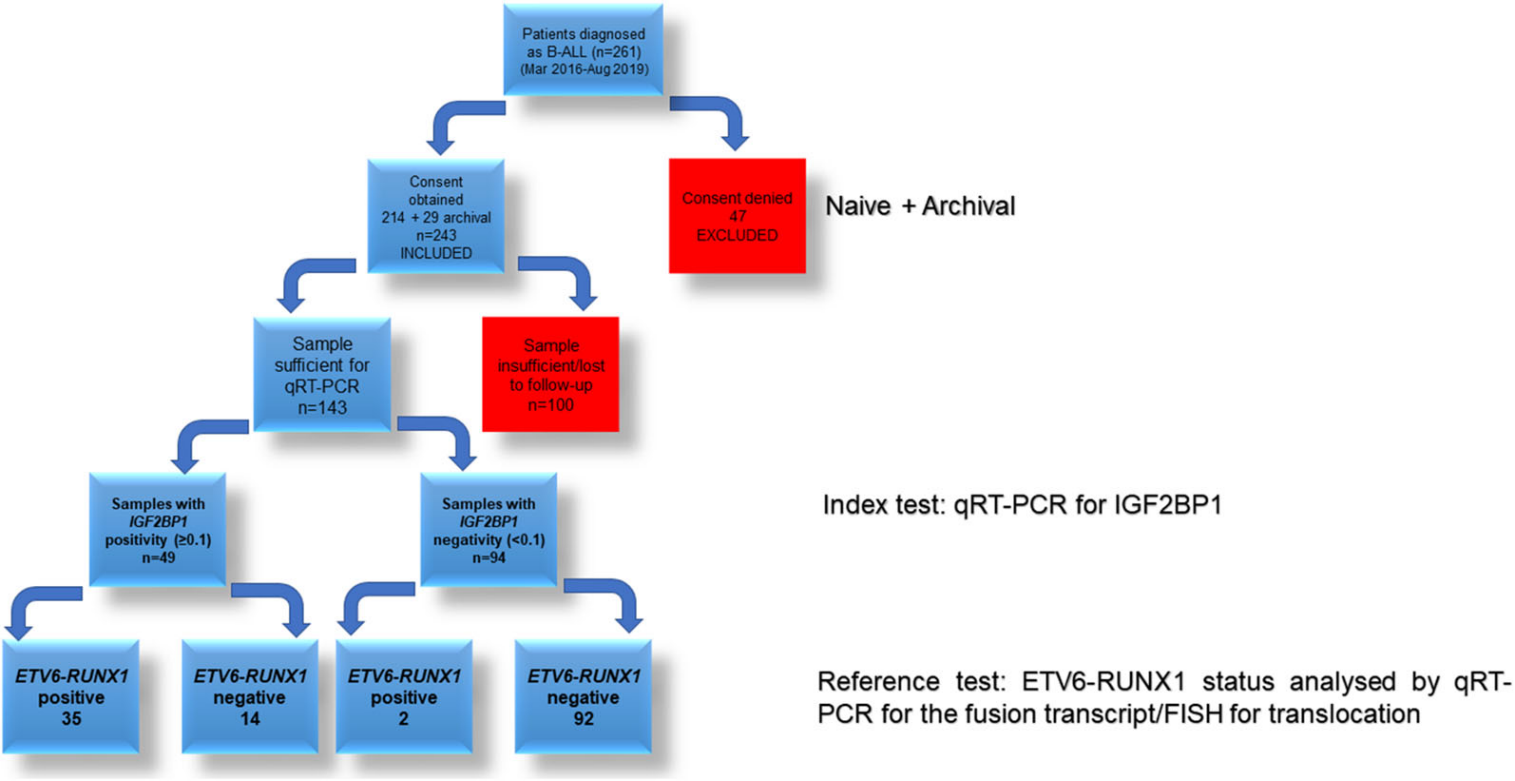
STARD diagram showing flow of participants through the study.

The potential of *IGF2BP1* expression to identify the *ETV6-RUNX1* translocation was studied using an ROC curve. *ETV6-RUNX1* positivity confirmed either by FISH for the t(12;21) translocation or PCR for *ETV6-RUNX1* fusion transcript or by both (**Figure 3C**) was utilized as the reference or gold standard (STARD flowchart in **Figure 4**). The area under the curve was 0.91 having a sensitivity of 95% and specificity of 86%, with a cut-off of >0.1 with respect to *RNA Pol II*. Various papers have previously identified *MYC* as a known target of IGF2BP1 (24, 31, 32). Interestingly, *MYC* expression showed significant downregulation in the *ETV6-RUNX1* positive group (**Figure 3D**), contradictory to the existing literature which suggests a *MYC* stabilizing role for IGF2BP1 (24). This might imply the tissue-specific function of IGF2BP1. ROC curves also suggested that reduced *MYC* expression, with a cut-off value of <0.63 with respect to *RNA Pol II*, has the potential to be used as a diagnostic marker to detect *ETV6-RUNX1* translocation, albeit with a lesser sensitivity (73%) and specificity (65%) than *IGF2BP1* expression (**Figure 3E**).

**Figure 5:**
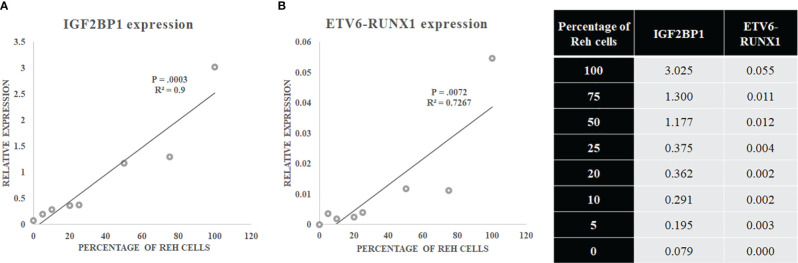
**(A)** IGF2BP1 and **(B)** ETV6-RUNX1 expression in a mixture of Reh (corresponding to blasts) and PBMCs.

The sensitivity of *IGF2BP1* expression to identify this translocation was compared against studying the expression of the *ETV6-RUNX1* fusion mRNA itself. Varying mixtures of Reh (*ETV6-RUNX1* translocation positive cell line: representing the blasts) and control PBMCs (no *ETV6-RUNX1*) were subjected to Real Time RT-PCR for *IGF2BP1* as well as *ETV6-RUNX1*. The correlation coefficient demonstrated the linearity and accuracy of *IGF2BP1* expression with an inferior correlation seen for *ETV6-RUNX1* expression. While the expression of both *ETV6-RUNX1* and *IGF2BP1* correlated significantly with the blast percentage, the relative expression of *ETV6-RUNX1* was much lower than *IGF2BP1* levels contributing to the higher sensitivity of *IGF2BP1* expression (**Figures 5A, B**).

### Validating the Efficacy of Putative IGF2BP1 Targets in Diagnosing ETV6-RUNX1 Positive Leukemia

Since IGF2BP1 is an RNA binding protein, we next proceeded on elucidating its cognate targets. To identify *ETV6-RUNX1* positive B-ALL specific putative targets of IGF2BP1, we compared three high-throughput gene expression datasets from published articles:

1. Microarray analysis of *ETV6-RUNX1* translocation positive patients (n = 4) compared to normal CD-19 positive B-cells from healthy donors (n = 2) (33);
2. Differential gene expression analysis among subtypes of B-ALL comprising of *ETV6-RUNX1*, *E2A-PBX1*, and *MLL* positive patients (n = 44) GSE65647 (34).
3. MILE (Microarray Innovations in LEukemia) study of B-ALL patient samples (n= 3242) (35, 36).

The first study (33) compared the gene expression levels of *ETV6-RUNX1* positive patients’ bone marrow with respect to the MACS sorted CD19 + B cells’ RNA using a microarray. We identified the top 100 overexpressed genes from this differentially expressed list. These are the genes which are specifically overexpressed due to the presence of the *ETV6-RUNX1* fusion protein.

The second dataset (34) compared the gene expression pattern between patient samples with three different translocations in B-ALL: *ETV6-RUNX1*, *E2A-PBX1*, and *MLL* translocations by microarray. From this data, we compared the three groups among each other. The genes specifically overexpressed in the comparison between these two different groups [*ETV6-RUNX1* vs. *E2A-PBX1 (203 genes)*, *ETV6-RUNX1* vs. *MLL (329 genes)*] were identified. These are the *ETV6-RUNX1* positive B-ALL specific genes from this study. Dataset 1 and 2 were overlapped to identify common genes.

The third dataset we used is from the MILE (Microarray Innovations in Leukemia) study (35, 36) which was used for corroboration. We used this study to check the expression of all the common genes from Dataset 1 and 2. We then selected only those genes whose expression levels were high in the *ETV6-RUNX1* cohort in the MILE study.

Using the three datasets, we had constructed a list of genes commonly overexpressed in *ETV6-RUNX1* positive B-ALL. We then intersected this list with the dataset from an eCLIP experiment done for IGF2BP1 in K562 cell line (37). CLIP (Cross-linked Immunoprecipitation) is a technique where cells are UV-crosslinked, pulled down with an IGF2BP1 specific antibody followed by RNA isolation and sequencing. The peaks from this dataset were visualized on the UCSC browser. The binding of IGF2BP1 to its targets was quantified as weak (+), medium (++) or strong (+++/++++).

**Figure 6:**
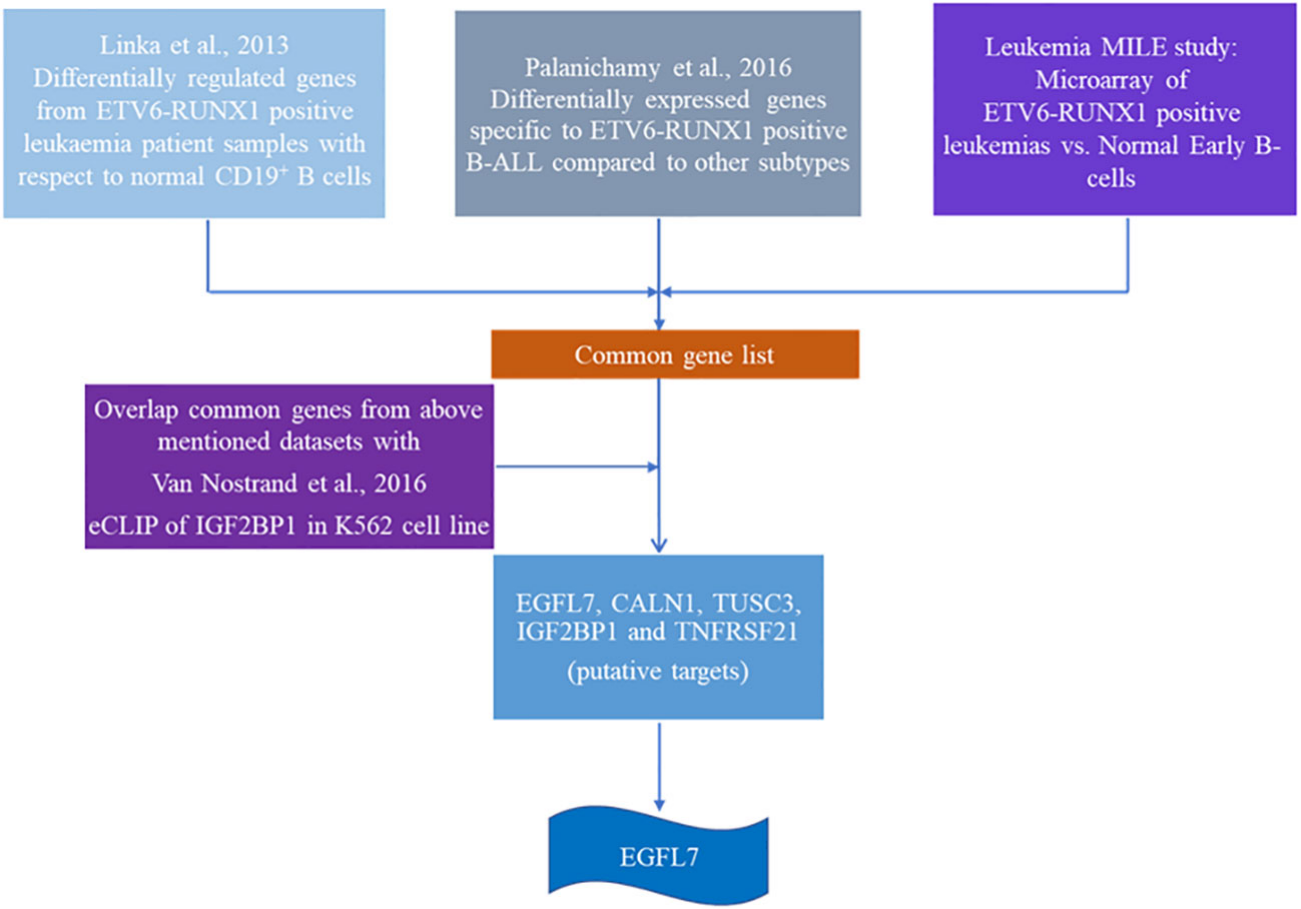
Schematic showing the algorithm used to identify putative targets of IGF2BP1 in ETV6-RUNX1 positive B-ALL.

The final list consisted of only 12 such genes which we further segregated based on their known function and putative role in any hematological malignancy (**Figure 6** and **Table 3**). Out of these 12 genes, *EGFL7*, *CALN1*, *TUSC3* and *TNFRSF21* were the top genes that were found to be highly expressed (>2 log fold change) in *ETV6-RUNX1* positive group in comparison to *MLL* and *E2A-PBX1* positive groups as well as were bound strongly by IGF2BP1 from the eCLIP dataset. *CALN1* has previously known to be overexpressed in *ETV6-RUNX1* leukemia but its exact function is still unknown (38), while *TUSC3* is reported to be a tumor suppressor gene (39). Among these gene, *EGFL7* has been shown to play a role previously in the pathogenesis of Acute Myeloid Leukemia (AML) and hematopoiesis and is also known to be overexpressed in *ETV6-RUNX1* leukemia (40, 41). Hence, we tested the potential of using *EGFL7* expression to diagnose the *ETV6-RUNX1* translocation.

**Table 3:** List of putative IGF2BP1 targets.

List of genes with IGF2BP1 binding and overexpression in ETV6-RUNX1 positive B-ALL							
Gene	ETV6-RUNX1 Vs. CD19+ve	Leukemia MILE study		Microarray data log FC			eCLIP of IGF2BP1
	mRNA Fold Change	E6R1+ B-ALL	Normal EARLY B cell	E6R1 Vs. MLL	E2A-PBX Vs. MLL	E6R1 Vs. E2A-PBX	BINDING
**IGF2BP1**	59.39268	High (+++)	Low	5.7	No change	6.17	++
**EGFL7**	88.58453	High (+++)	Low	2.5	−1.4	3.9	+++
**CALN1**	65.20498	High (+++)	NA	2.99	No change	3.08	++
**TUSC3**	65.20426	High (+++)	High (+)	3.12	No change	3.37	++
**TNFRSF21**	59.27442	High (+++)	High (+)	3.02	No change	3.13	+
**FSCN1**	85.27267	High (++)	High(+++)	1.06	−2.8	3.8	+++
**MDK**	79.06593	High (+++)	Low	4.93	3.5	1.42	+++
**MYO18B**	70.15012	High (+++)	NA	1.64	No change	No change	++
**SPTA1**	100	High (++)	Low	1.9	No change	1.8	+
**TMEM136**	100	High (+++)	High (+)	4.04	No change	No change	+
**DYRK3**	99.44952	High (+++)	Low	2.42	2.62	No change	+++
**SOCS2**	95.09744	High (+++)	High (+)	−1.14	−6.5	5.4	+++

**Figure 7:**
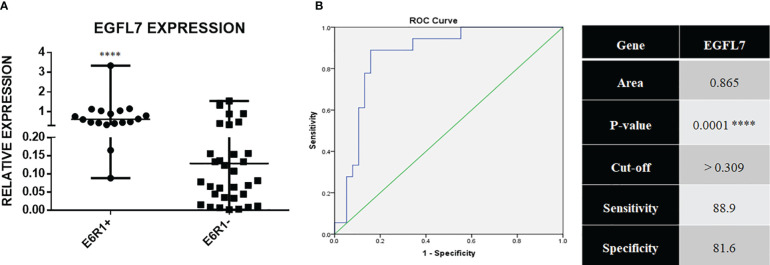
**(A)** Real time expression data showing EGFL7 overexpression in ETV6-RUNX1 translocation positive patients (N = 18) and ETV6-RUNX1 translocation negative patients (N = 38); and **(B)** ROC curve for EGFL7 ****p < 0.001.

**Figure 8:**
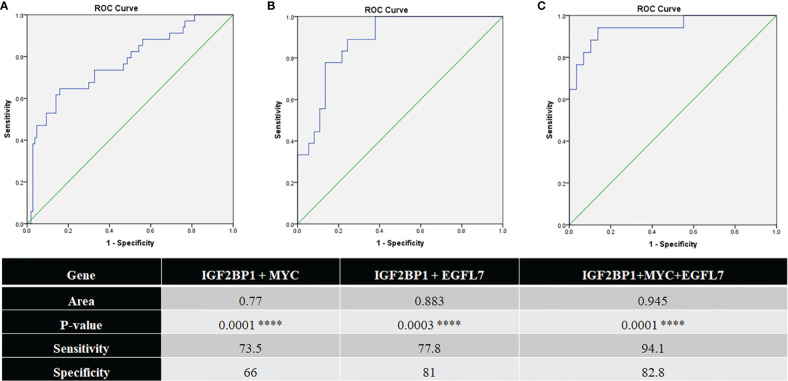
ROC curves for **(A)** IGF2BP1 + MYC (N = 141; pos = 34, neg = 107); **(B)** IGF2BP1 + EGFL7 (N = 55; pos = 18, neg = 37); and **(C)** IGF2BP1 + MYC + EGFL7 (N = 46; pos = 17, neg = 29) comparing them to FISH/RT-PCR for ETV6-RUNX1 ****p < 0.001.

*EGFL7* mRNA was also found to be significantly over expressed in the *ETV6-RUNX1* translocation positive group in our cohort (**Figure 7A**). ROC curves for *EGFL7* also showed potential of being a biomarker to diagnose *ETV6-RUNX1* translocation but still slightly inferior to *IGF2BP1* expression (AUC = 0.865 with a sensitivity of 88.9% and specificity of 81.6%, with cut-off **>**0.309 with respect to *RNA Pol II*) (**Figure 7B**). We tried using the expression levels of *IGF2BP1*, *MYC*, and *EGFL7* in various combinations to study their diagnostic efficiency. All the combinations had a good AUC but *IGF2BP1* expression alone was the best with the highest sensitivity and specificity at a relative expression cut-off **>**0.1 (**Figures 8A–C**).

**Figure 9:**
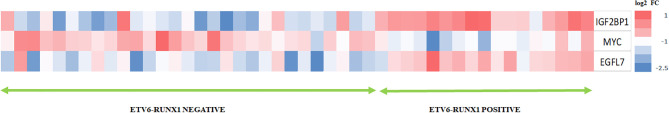
Heat map showing the expression of IGF2BP1, MYC, and EGFL7 in ETV6-RUNX1 translocation positive patients with respect to ETV6-RUNX1 translocation negative patients (N = 46).

**Figure 10:**
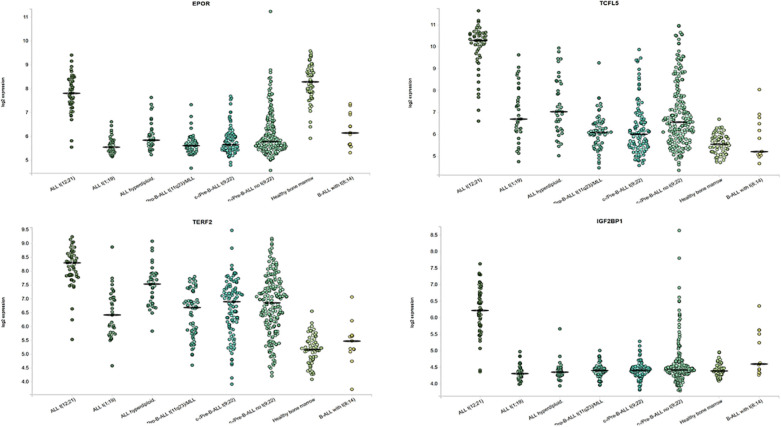
Expression of ETV6-RUNX1 positive B-ALL specific genes and IGF2BP1 from the MILE study. ALL with hyperdiploid karyotype (n=40), ALL with t(1;19) (n=36), ALL with t(12;21) (n=58), Mature B-ALL with t(8;14) (n=13), c- ALL/Pre-B-ALL with no t(9;22) (n=237), c- ALL/Pre-B-ALL with t(9;22) (n=122), Non-leukemic Healthy bone marrow (n=73), Pro-B-ALL with t(11q23)/MLL (n=70).

The heatmap showing the expression of *IGF2BP1*, *MYC*, and *EGFL7* in *ETV6-RUNX1* translocation positive and negative patients highlights the discriminatory power of *IGF2BP1* to identify this translocation (**Figure 9**). Previous studies have identified various genes including *EPOR*, *TCFL5*, and *TERF2* to be specifically overexpressed in *ETV6-RUNX1* leukemia (38). We compared the expression of *IGF2BP1* as well as these genes in ALL patient samples from the publicly available Leukemia MILE study (36) on the BloodSpot database (35). Although all these genes are overexpressed in *ETV6-RUNX1* positive B-ALL, it is evident that *IGF2BP1* expression stands out in being extremely specific to this subtype (**Figure 10**).

## Discussion

Routine diagnostic techniques used in ALL diagnosis and management include flowcytometry (for immunophenotyping), RT-PCR, Karyotyping, and FISH. FISH is the gold standard technique which is commonly used to diagnose the presence of leukemic translocations and it has distinct advantages (21, 42). The average turnaround time (TAT) for FISH is 3 days (43) and cytogenetics is 7 days (44) in standard laboratories. We have investigated the utility of studying the expression of *IGF2BP1*, an RNA binding protein known to be overexpressed in *ETV6-RUNX1* leukemia, by qPCR, to be used as a cheaper and quicker alternative to diagnose the presence of the translocation. This would facilitate risk stratification using low amounts of patient bone marrow sample. This would be especially useful in pediatric patients where the sample obtained during the bone marrow aspiration is not sufficient enough to even complete the panel of routine diagnostic tests (45). If utilized, the TAT for gene expression analysis is ~6 h and can be done easily in house without incurring extra cost to the patient. Based on the sensitivity and specificity of the gene as demonstrated in the results, we could potentially add the qPCR for *IGF2BP1* as a diagnostic marker for the presence of *ETV6-RUNX1* translocation. This study has utilized the most commonly observed sentinel translocations (*BCR-ABL1*, *E2A-PBX1*, *MLL-AF4*). This study has not addressed the newer gene expression based minor subtypes including the “*ETV6-RUNX1*-like” leukemia which would be part of the “no known sentinel translocations” group.

This is the first study to analyze the utility of gene expression analysis of *IGF2BP1* for the diagnosis of *ETV6-RUNX1* positive B-ALL. It is also the first time that such an exercise has been carried out in an Indian patient cohort of over 100 patients. Since IGF2BP1 is an RNA binding protein, we have used data mining from public high throughput datasets to identify its targets in B-ALL. We have utilized three datasets to identify an *ETV6-RUNX1* specific gene signature which was then overlapped with an eCLIP dataset of IGF2BP1. The overlapped dataset gave us a novel list of *ETV6-RUNX1* specific genes bound and stabilized by IGF2BP1.

From this list we have further characterized two genes (*EGFL7* and *MYC*) for their sensitivity and specificity in identifying the translocation. Both these genes were also found to have the potential to be used as biomarkers to identify the translocation. However, *IGF2BP1* mRNA expression by RT qPCR was found to be an excellent candidate to be used as a biomarker to diagnose *ETV6-RUNX1* translocation showing 95% sensitivity and 86% specificity at a relative expression cut off of 0.1. *IGF2BP1* expression in the bone marrow as well as peripheral blood can be utilized as a rapid test to diagnose the presence of the *ETV6-RUNX1* translocation in the future.

This increase in *IGF2BP1* expression might be due to the loss of ETV6 which might be repressing IGF2BP1 or the presence of the *ETV6-RUNX1* fusion protein or it might be a combination of both factors. Interestingly, the de-repression of *IGF2BP1* in *ETV6-RUNX1* positive ALL is so strong that its gene expression is ~100 fold higher than the expression of the *ETV6-RUNX1* fusion RNA. This contributes to the extremely high sensitivity and specificity of this assay. Future work could also involve identifying the mechanistic role of IGF2BP1 in leukemogenesis and developing it into a therapeutic target in this particular subset of B-ALL.

## Data Availability Statement

The original contributions presented in the study are included in the article/supplementary material. Further inquiries can be directed to the corresponding author.

## Ethics Statement

The studies involving human participants were reviewed and approved by Institutional Ethics Committee, All India Institute of Medical Sciences, New Delhi, India. Written informed consent to participate in this study was provided by the participants’ legal guardian/next of kin.

## Author Contributions

GS, EB, and TN carried out the experiments. AJ, GS, JS, and EB contributed to sample collection and analysis. SB and AC provided patient samples and clinical data. GS and JP wrote the manuscript with inputs from AS, SB, PC, and AC. JP conceived the study. All authors contributed to the article and approved the submitted version.

## Funding

This work is being supported by the Wellcome Trust/DBT India Alliance Early Career Fellowship to JP (IA/CPHE/15/1/502050). GS is supported by DBT Senior Research Fellowship as well as SERB Overseas Visiting Doctoral Research Fellowship. JS is being supported by a DBT Senior Research Fellowship and AJ by a CSIR Senior Research Fellowship. We thank Dr Kalaivani for her help with the statistical analysis. We thank all our collaborators for vibrant discussions regarding the experiments and data.

## Conflict of Interest

The authors declare that the research was conducted in the absence of any commercial or financial relationships that could be construed as a potential conflict of interest.
